# The mirror like expression of genes involved in the FOXO signaling pathway could be effective in the pathogenesis of human lymphotropic virus type 1 (HTLV-1) through disruption of the downstream pathways

**DOI:** 10.1186/s13104-023-06423-x

**Published:** 2023-07-17

**Authors:** Sahar Yaslianifard, Monireh Movahedi, Somayeh Yaslianifard, Sayed‑Hamidreza Mozhgani

**Affiliations:** 1 Department of Biochemistry, Faculty of Biological Sciences, NorthTehran Branch, Islamic Azad University, Tehran, Iran; 2 Department of Microbiology and Virology, School of Medicine, Alborz University of Medical Sciences, Karaj, Iran; 3 Dietary Supplements and Probiotic Research Center, Alborz University of Medical Sciences, Karaj, Iran; 4 Non-Communicable Diseases Research Center, Alborz University of Medical Sciences, Karaj, Iran

**Keywords:** Human T‑lymphotropic virus type 1, Adult T‑cell leukemia/lymphoma, HTLV‑1 associated myelopathy/tropical spastic paraparesis, Pathogenesis

## Abstract

**Objectives:**

Human lymphotropic virus type 1 (HTLV-1) is the cause of two major diseases, ATLL and HAM/TSP in a percentage of carriers. Despite progress in understanding the pathogenesis of these two diseases, the exact pathogenesis mechanism is still not well understood. High-throughput technologies have revolutionized medical research. This study aims to investigate the mechanism of pathogenesis of these two diseases using the results of high-throughput analysis of microarray datasets.

**Results:**

A total of 100 differentially expressed genes were found between ATLL and HAM/TSP. After constructing protein-protein network and further analyzing, proteins including ATM, CD8, CXCR4, PIK3R1 and CD2 were found as the hub ones between ATLL and HAM/TSP. Finding the modules of the subnetwork revealed the enrichment of two common pathways including FOXO signaling pathway and Cell cycle with two common genes including ATM and CDKN2D. Unlike ATLL, ATM gene had higher expressions in HAM/TSP patients. The expression of CDKN2D was increased in ATLL patients. The results of this study could be helpful for understanding the pathogenic mechanism of these two diseases in the same signaling pathways.

**Supplementary Information:**

The online version contains supplementary material available at 10.1186/s13104-023-06423-x.

## Introduction

Human lymphotropic virus type I (HTLV-1) is a member of the Retroviridae family, which is in the genus Deltavirus. This virus does not cause clinical symptoms in 95% of infected people, but the remaining 5% progress to adult T-cell leukemia/lymphoma (ATLL) or/and (HTLV-1)-associated myelopathy/tropical spastic paraparesis (HAM/TSP). It is estimated that between 15 and 20 million people in the world are infected with the virus. This virus is more prevalent in Japan, Iran, South Africa, and South America [1–3].

High-throughput studies provide possibility to find the simultaneous expression of thousands of gene. The further analyses help understand the affected pathways resulting from the changes in the expression value of the involved genes. In these types of studies, the whole transcriptome is evaluated and the differentially expressed genes (DEGs) or co-expressed genes could be identified. Therefore, the molecular disorders leading to development of a disease could be more clearly understood [4–7]. In the HTLV-1 associated diseases (ATLL and HAM/TSP), in addition to the viral mechanisms, diverse cellular signaling pathways are also involved [8, 9]. Common genes with different and even reverse expressions are involved in the development of these diseases with two different clinical signs. It is not yet clear why some people develop these diseases and others do not. A series of previous high-throughput studies compared ATLL and HAM/TSP with healthy groups as well as asymptomatic carriers (ACs), but few studies have compared these two diseases with each other [6, 10–14]. Since the molecular mechanism leading to the progression of the ATLL and HAM/TSP has not yet been well clarified, this study aims to identify signaling pathways and genes with significant expression differences between ATLL and HAM/TSP patients using the analysis of the microarray data. The identified genes are introduced as the possible molecular players implicated in the fate of HTLV-1 infection toward two possible diseases.

## Materials and methods

### Gene expression microarray dataset

In this study, gene expression profile with accession number GSE19080 and platform number 9686 through gene expression omnibus public repository (www.ncbi.nlm.nih.gov/ geo) was used. The authors performed the microarray experiments using the human ImmuneArray cDNA array. This dataset includes a total of 38 samples, of which 7 samples belong to ATLL patients, 12 samples belong to HAM/TSP patients, 11 samples included healthy carriers and 8 samples belong to normal people. In accordance with the objectives of this survey, only ATLL and HAM/TSP samples were used.

### Gene expression dataset and differential expression analysis

The GEO2R (http://www.ncbi.nlm.nih.gov/geo/geo2r/) was employed to perform log2 transformation, recognition of DEGs, and calculation of fold change (FC). In addition, differential expression analysis between two groups of ATLL and HAM/TSP patients was visualized using package pheatmap in R 3.2.5. Based on variance of expression, first 100 genes of each group with highest values were selected to generate heatmap plot.

### Protein-protein interaction network (PPIN)

The online STRING database was performed to construct the PPIN. The interactions presented in this database are based on genomic context, high-throughput experiments, co-expression, and previous knowledge (databases and text-mining) [15]. The combined score higher than 0.4 was considered as cut- off to analyze the PPIN.

### Reconstruction of PPIN and centrality analysis

The PPIN obtained from the STRING database was analyzed using the Network Analyzer app in the Cytoscape (3.5.1) software, and the degree centrality criteria was calculated. These criteria reflect the amount of extension from each node to other accessible nodes. Using the information obtained from these calculations, the nodes with the highest score in the degree value were used to construct the subnetwork. Gephi version 0.9.1, an open-source network visualization and manipulation software was used for further analysis and visualization of the obtained subnetwork [16].

### The identification of functional modules and gene enrichment analysis

To divide the obtained subnetwork into the corresponding modules, the fast-unfolding clustering algorithm was executed in Gephi version 0.9.1. Then, each of the obtained modules was enriched using the Enricher web tool. Significantly Kyoto Encyclopedia of Genes and Genomes (KEGG) pathways terms were taken based on the top ten combined scores.

### Study participants and quantitative real-time PCR

For data validation quantitative real-time polymerase chain reaction (RT-qPCR) was performed on the cDNA samples of 10 HAM/TSP and 10 ATLL patients to measure the expression of ATM, CDKN2D and RPLP0 using the SYBR Green qPCR Master Mix (TaKaRa, Otsu, Japan). The nucleotide sequences of designed primers were as follow: forward primer of ATM 5- CTGCTGCCGTCAACTAGAAC-3, reverse primer of ATM 5-AGGCTTGTGTTGAGGCTGAT-3, forward primer of CDKN2D 5-TGATGTCAACGTGCCTGATG-3’, reverse primer of CDKN2D 5- AGCTCCAAGGGTGTGAGAC-3. RPLP0 was utilized as a housekeeping gene in order to normalize the mRNA expression levels, as well as control error between samples [17]. The relative expressions were calculated based on the expression ratio of ATM/RPLP0 and CDKN2D /RPLP0. Data were analyzed by Prism GraphPad Software Version 8.0.2 (GraphPad software, Inc., San Diego, CA, USA). This study was approved by the medical ethics committee of Alborz University of Medical Sciences (IR.ABZUMS.REC.1398.108).

## Results

### Differential expression analysis (DEGs)

**Fig. 1:**
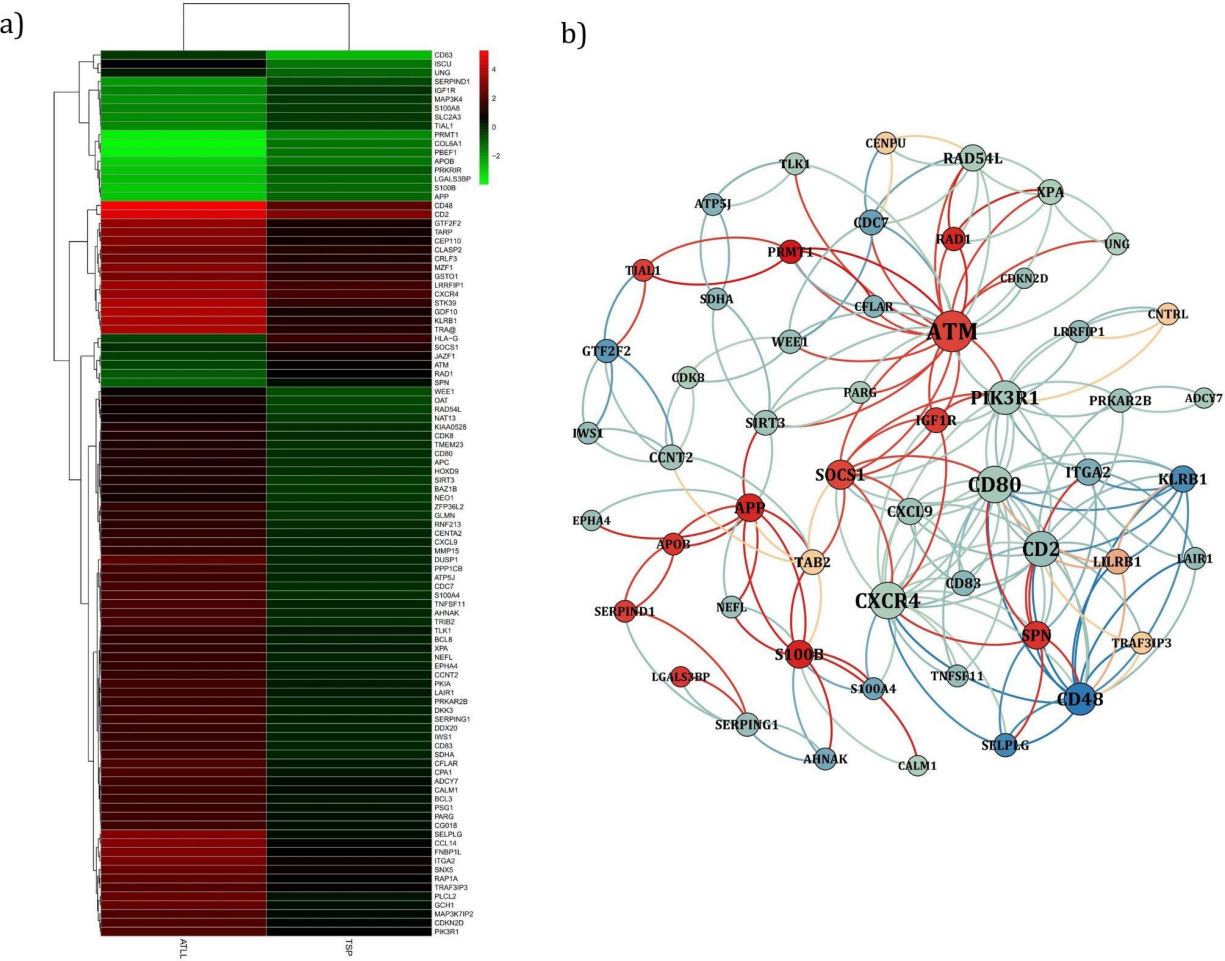
(**a**) The heatmap of the first 100 genes according to the variance of gene expression among two groups of patients. The color gradient indicates the interval between the highest (red) and the lowest (Green) gene expression. (**b**) The PPINs between the recognized hub DEGs of ATLL vs. HAM/TSP patients, the node size is indicative of degree of nodes and the color gradient indicates the interval between the highest (red) and the lowest (indigo) gene expression

The heatmap plot of the first 100 genes belonging to ATLL and HAM/TSP samples were constructed. To this purpose, the mean expression level of genes was calculated for each group. Then, the top 100 genes among two sample groups were selected based on variances difference. In the constructed heatmap, the color gradient from red to green indicates the expression level of genes from the highest to the lowest expression level. The down-regulated genes are identified as green and up-regulated genes are specified as red (Fig. 1a). Table 1 shows DEGs and their expression values by LogFC. A positive LogFC indicates an increase in gene expression in HAM/TSP patients compared to ATLL patients. The statistically significant value between the two mentioned groups is showed considering adjusted p < 0.05. value. The five genes with the highest expression value were PRMT1, S100B, RAD1, APP, and SPN, and the five genes with the highest decrease in the expression value were CD48, SELPLG, KLRB1, GTF2F2, and CDC7.

**Table 1:** List of the upregulated and downregulated hub genes in each group. The value of gene expression is showed by LogFC. A positive LogFC indicates an increase in gene expression in HAM/TSP patients compared to ATLL patients. The statistically significant value between the two mentioned groups is showed by adjusted P. Value

Symbol	logFC	adj.P.Val	Symbol	logFC	adj.P.Val
**ATM**	1.43	0.00021	**RAD1**	1.71	0.00905
**CD80**	-1.51	0.00001	**GTF2F2**	-2.35	0.00301
**CXCR4**	-1.41	0.04006	**SELPLG**	-2.65	0.00006
**PIK3R1**	-1.52	0.01943	**S100A4**	-2.11	0.00008
**CD2**	-1.68	0.00047	**TIAL1**	1.46	0.01677
**CD48**	-2.91	0.00001	**ADCY7**	-1.45	0.00244
**SOCS1**	1.41	0.01001	**AHNAK**	-2.07	0.00039
**S100B**	1.74	0.00130	**APOB**	1.56	0.00749
**APP**	1.71	0.00126	**SERPIND1**	1.47	0.00011
**CXCL9**	-1.57	0.00151	**EPHA4**	-1.60	0.00123
**SPN**	1.60	0.00300	**NEFL**	-1.61	0.00876
**ITGA2**	-1.98	0.00484	**UNG**	-1.40	0.00082
**SIRT3**	-1.59	0.00032	**CFLAR**	-1.89	0.00013
**RAD54L**	-1.47	0.00014	**TLK1**	-1.44	0.00124
**KLRB1**	-2.56	0.00001	**PARG**	-1.49	0.00554
**IGF1R**	1.50	0.00013	**CDKN2D**	-1.65	0.00288
**CDC7**	-2.19	0.00008	**ATP5J**	-1.88	0.00008
**XPA**	-1.42	0.00013	**CALM1**	-1.42	0.00406
**SDHA**	-1.78	0.00124	**IWS1**	-1.70	0.00021
**CCNT2**	-1.57	0.00013	**CDK8**	-1.39	0.00008
**LILRB1**	0.35	0.26656	**TRAF3IP3**	-1.47	0.00710
**CD83**	-1.80	0.00001	**LAIR1**	-1.71	0.00171
**PRKAR2B**	-1.60	0.00018	**TNFSF11**	-1.71	0.00171
**SERPING1**	-1.66	0.00008	**LRRFIP1**	-1.71	0.00059
**PRMT1**	1.88	0.06741	**LGALS3BP**	1.54	0.01576
**WEE1**	-1.64	0.00022			

### Construction of PPI network and extraction of the PPI subnetwork

The PPIN was used in order to inspect the stock relationship between DEGs. In order to construct the PPIN with the highest degree of connectivity, the primary network was constructed using STRING database. Afterward, the subnetwork including the proteins with the highest degree of connectivity in the primary network was build and visualized (Fig. 1b). The subnetwork consisted of 54 nodes and 212 edges for ATLL vs. HAM/TSP patients. In the obtained subnetwork, the size of the nodes indicates the degree of each node and the color of the nodes shows the expression level of each protein. The red color is indicative of the proteins with higher expression and the blue color is representative of the proteins with higher negative expression. The five proteins with the highest degree value are ATM, CD8, CXCR4, PIK3R1 and CD2.

### The identification of functional modules and pathway enrichment analysis

**Fig. 2:**
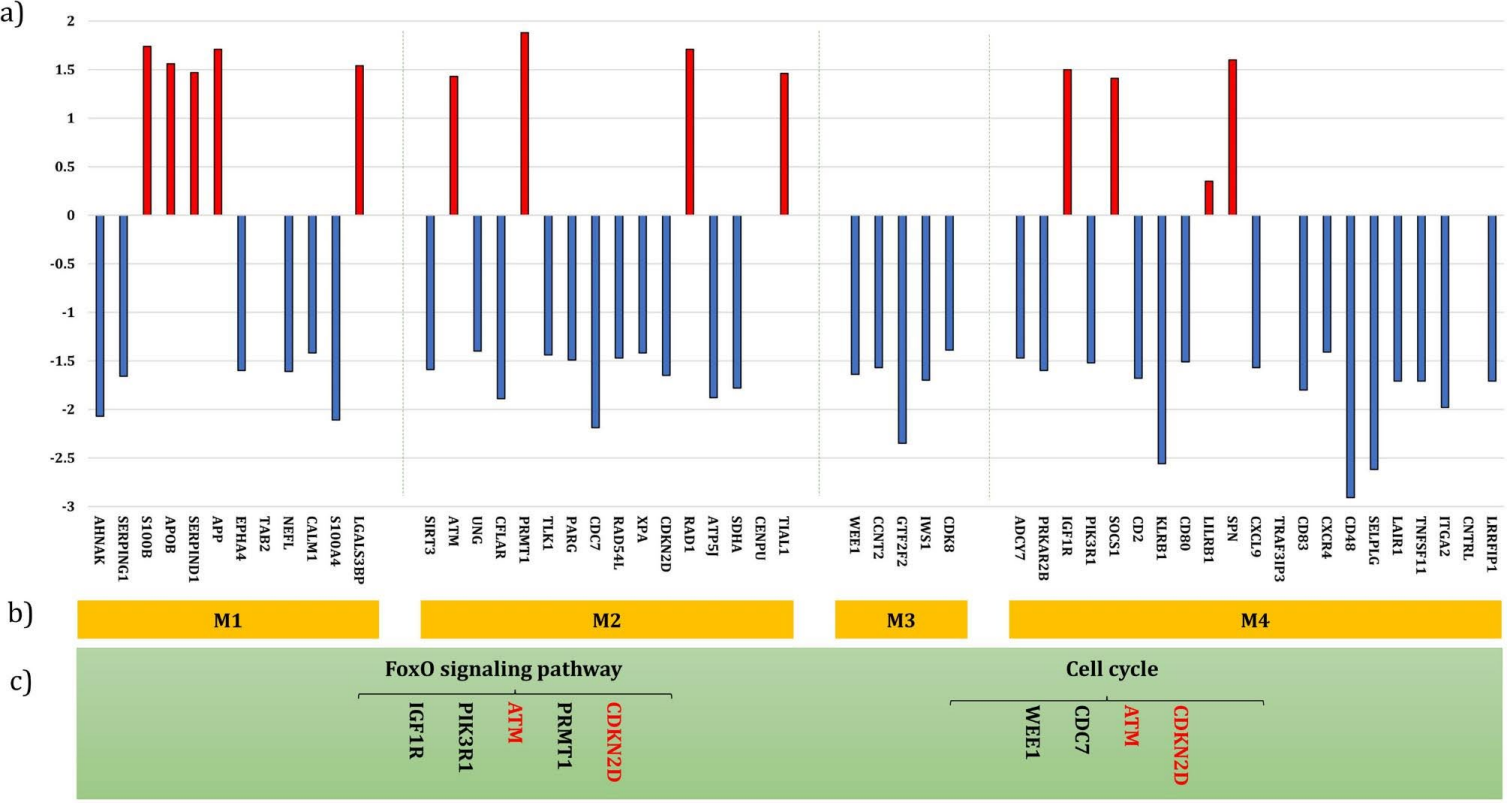
**a** and **b**) The 4 functional modules identified from constructed subnetwork of ATLL vs. HAM/TSP patients. The upregulated and downregulated genes are tagged by color. **c**) The most significant GO signaling pathways in top ranks of combined score were FOXO signaling pathway and Cell cycle. Further gene enrichment analysis showed that ATM and CDKN2D genes are common in both pathways. Unlike ATLL, ATM gene had higher expression in HAM/TSP patients. The expression of CDKN2D was increased in ATLL patients.

To find the modules of the subnetwork, the fast-unfolding clustering algorithm was applied. As a result, four modules (M1, M2, M3, M4) were obtained. The up-regulated and down-regulated genes are tagged by color. To undertake enrichment analysis among the detected modules, EnrichR web-based tool was used. The most significant GO signaling pathways in top ranks of combined score were FOXO signaling pathway and Cell cycle (Fig. 2a,b**).**

### The identification of common genes

Figure 2c shows the involve genes in the abovementioned pathways as well as common genes between them. ATM and CDKN2D genes are common in both pathways. Unlike ATLL, ATM gene had higher expressions in HAM/TSP patients but the expression of CDKN2D was increased in ATLL patients.

### ATM and CDKN2D gene expression

The mean ATM gene expression in HAM/TSP and ATLL reported as 1.21 ± 0.24 and 0.35 ± 0.17, respectively. A significant increase observed in HAM/TSP compared to ATLL (*P* = 0.004). The mean CDKN2D gene expression in HAM/TSP participants and ATLL illustrated to be 0.26 ± 0.25 and 1.40 ± 0.16, respectively. In ATLL, CDKN2D expressed at a significantly higher level than in HAM/TSP individuals (*P* = 0.003). (Supplementary 1).

## Discussion

The obtained results in this study, which is based on high-throughput analysis of substantial data of the whole transcriptome, revealed that PRMT1, S100B, RAD1, APP, and SPN genes of patients with HAM/TSP have the highest expression value. Also, CD48, SELPLG, KLRB1, GTF2F2, and CDC7 genes have the lowest expression in patients with HAM/TSP. One of the advantages of this study, which has rarely been mentioned in previous studies [8, 9], is the simultaneous comparison of genes with differential expression in patients with HAM/TSP compared to patients with ATLL. This study specifically identifies that if a gene has an increased expression in a HAM/TSP disease, it has a decreased expression in ATLL disease and vice versa. This inverse or mirror like expression can be of particular interest to researchers who work on the molecular pathogenesis of the mentioned associated diseases.

In this study based on variance of expression, the first 100 genes with highest values were selected and visualized by heatmap. These 100 genes were further analyzed and their interaction whit each other were shown in a PPIN. The five genes with the highest degree value in the drawn PPIN were ATM, CD8, CXCR4, PIK3R1 and CD2 that introduced as hub genes in the PPIN. Therefore, regardless of the increase or decrease in the expression, are of potential importance for further investigation.

In this study, four modules were obtained from the bioinformatic analysis of PPIN, and each module contains its own genes depending on the physical interaction of them together. Enrichment of genes in the obtained modules determined that the cell cycle signaling pathway and the FOXO signaling pathway are common among them. It is interesting to note that ATM and CDKN2D genes are common in both pathways.

FOXO1 activation plays a role in cell cycle progression regulation. The transcription and half-life of cyclin-dependent kinase inhibitor p27KIP1 rises when FOXO1 is active and could impact cell cycle progression [18]. ATM serine/threonine kinase, symbol ATM, is a serine/threonine protein kinase that is recruited and activated by DNA double-strand breaks [19]. The activation of this protein plays an effective role in activation of the DNA damage checkpoint, leading to DNA repair, apoptosis or cell cycle arrest [20]. CDKN2D is a member of the INK4 family CKIs. This protein regulates the G1-to-S phase transition by specifically inhibiting the activity of CDK4 and CDK6 [21]. In previous studies, the role of this protein in the induction of malignancy has not been well clarified [22]. The results of this study can potentially confirm the role of this protein in the development of ATLL.

## Conclusion

In this study, it was found that dysregulation of FOXO signaling pathways and Cell cycle can be effective in the development of the ATLL or HAM/TSP diseases. In addition, it was found that ATM and CDKN2D genes are common in the mentioned pathways and their expression disorder is effective in the development of these diseases. The obtained results could be helpful for understanding the pathogenic mechanism of these two diseases in the same signaling pathways.

### Limitations

Further detailed studies help us understand other functions of the involved genes in the pathogenesis of HTLV-1.

## Electronic supplementary material

Below is the link to the electronic supplementary material.

**Supplementary 1**. The expression levels of ATM (a) and CDKN2D (B) in HAM/TSP and ATLL individuals.

## Data Availability

In this study, gene expression profile with accession number GSE19080 and platform number 9686 through gene expression omnibus public repository (www.ncbi.nlm.nih.gov/ geo) was used.

## Abbreviations

HTLV‑1: Human T‑lymphotropic virus type 1
HAM/TSP: HTLV‑1‑associated myelopathy/tropical spastic paraparesis
ATLL: Adult T‑cell leukemia/lymphoma
GEO: Gene Expression Omnibus
DEGs: Differentially expressed genes
STRING: Search Tool for the Retrieval of Interacting Genes
PPIN: Protein–protein interaction network
