# Supplementary material for: The mirror like expression of genes involved in the FOXO signaling pathway could be effective in the pathogenesis of human lymphotropic virus type 1 (HTLV-1) through disruption of the downstream pathways
Source: BMC Res Notes. 2023 Jul 17;16:147. doi: 10.1186/s13104-023-06423-x (PMC10353225; doi:10.1186/s13104-023-06423-x)
Supplement: Supplementary file 1 — Supplementary 1. The expression levels of ATM (a) and CDKN2D (B) in HAM/TSP and ATLL individuals. [file 13104_2023_6423_MOESM1_ESM.docx]

**Supplementary**


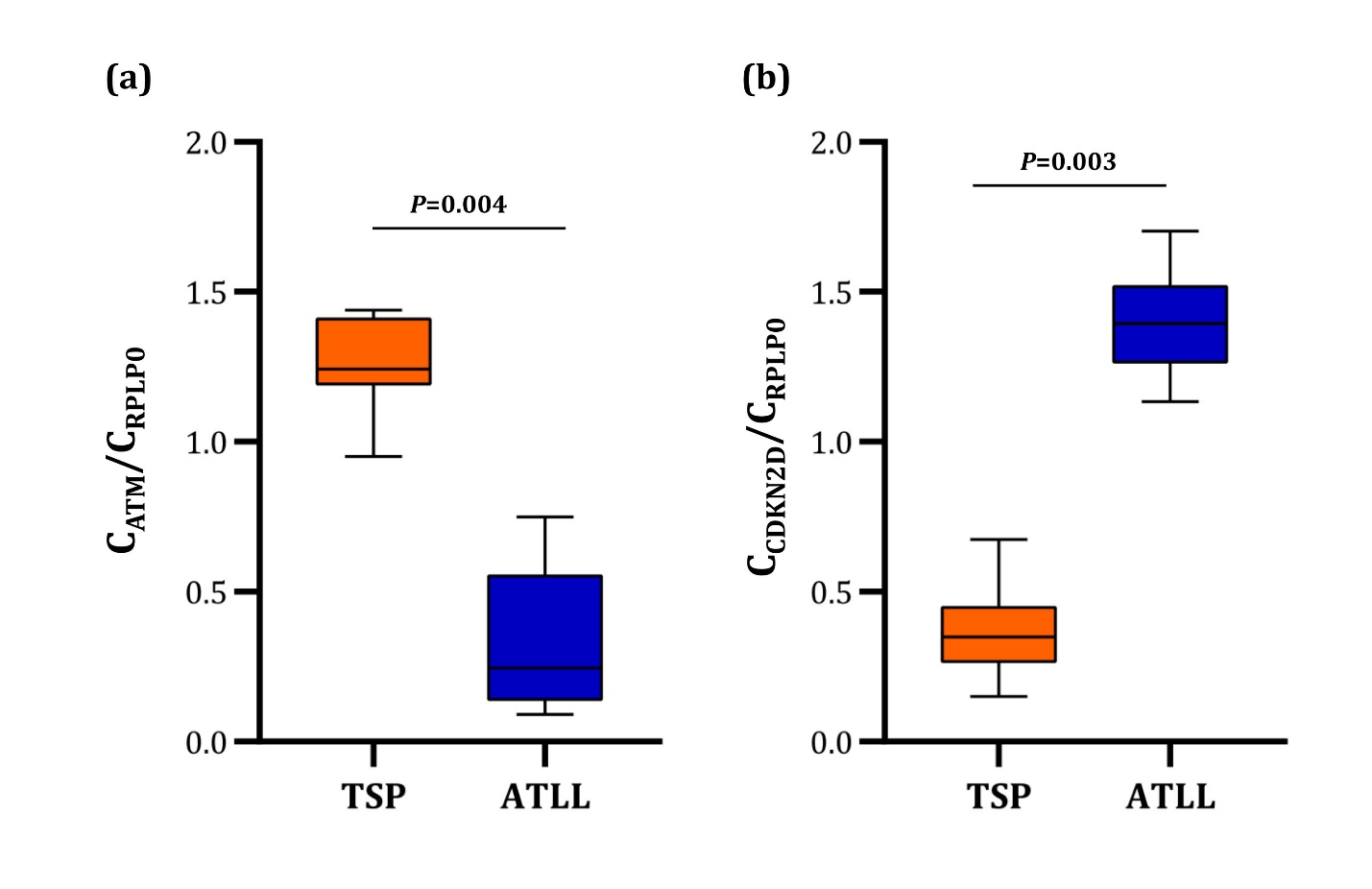


**Supplementary 1.** The expression levels of ATM (a) and CDKN2D (B) in HAM/TSP and ATLL individuals.
